# Opposing serial dependencies revealed for sequences of auditory emotional stimuli

**DOI:** 10.1177/03010066241235562

**Published:** 2024-03-14

**Authors:** Erik Van der Burg, Martijn Baart, Jean Vroomen, Huihui Zhang, David Alais

**Affiliations:** 1234 University of Amsterdam, Netherlands; 1190 Vrije Universiteit Amsterdam, Netherlands; 7899 Tilburg University, Netherlands; 12465 Peking University, Beijing, China; University of Sydney, Australia

**Keywords:** serial dependencies, emotions in speech, response bias, go–no-go task, audition

## Abstract

Our percept of the world is not solely determined by what we perceive and process at a given moment in time, but also depends on what we processed recently. In the present study, we investigate whether the perceived emotion of a spoken sentence is contingent upon the emotion of an auditory stimulus on the preceding trial (i.e., serial dependence). Thereto, participants were exposed to spoken sentences that varied in emotional affect by changing the prosody that ranged from ‘happy’ to ‘fearful’. Participants were instructed to rate the emotion. We found a positive serial dependence for emotion processing whereby the perceived emotion was biased towards the emotion on the preceding trial. When we introduced ‘no-go’ trials (i.e., no rating was required), we found a negative serial dependence when participants knew in advance to withhold their response on a given trial (Experiment 2) and a positive serial dependence when participants received the information to withhold their response after the stimulus presentation (Experiment 3). We therefore established a robust serial dependence for emotion processing in speech and introduce a methodology to disentangle perceptual from post-perceptual processes. This approach can be applied to the vast majority of studies investigating sequential dependencies to separate positive from negative serial dependence.

In recent years, the area of serial dependence has been a very active field of perceptual research. The important contribution of these studies has been to demonstrate that our perception of the world is not solely determined by current sensory input but also by stimuli from the immediate past (Kiyonaga et al., 2017; Pascucci et al., 2023). A way to measure such sequential dependencies is by presenting participants with sequences of brief stimuli, measuring their perception of each one, and then conducting an inter-trial analysis to see if perception of a given stimulus depends on the preceding one. For example, if this is done for a set of lines that varies in orientation over trials, there is a sequential dependency in which the perceived orientation of the current orientation is always slightly biased *towards* the previously presented orientation (Fischer & Whitney, 2014). As current perception tends to follow the previous percept, this is known as a positive (in some studies known as attractive) serial dependence. Serial dependence is effectively a short-term temporal averaging process, which improves the signal-to-noise ratio and serves to stabilise perception (Cicchini & Burr, 2018; Kiyonaga et al., 2017).

Positive serial dependence is a pervasive phenomenon in visual perception. It has been shown for perceptual judgements of a wide range of basic visual attributes, including motion direction (Alais et al., 2017), luminance (Fründ et al., 2014), orientation (Fischer & Whitney, 2014), as well as for many higher-level visual stimuli involving global processing, such as face perception (Liberman et al., 2014; Taubert, Alais, et al., 2016; Van der Burg et al., 2019), scene perception (Manassi et al., 2017), and even ratings of art works (S. Kim et al., 2019). In the auditory domain, work on positive serial dependence has been much less active but it has been demonstrated for auditory rate perception (Motala et al., 2020), pitch judgements (Arzounian et al., 2017) and judgements about the auditory duration (Li et al., 2023). How widely it may occur in auditory perception is still unknown. However, given that auditory input varies temporally on such a fine scale, the ‘temporal averaging’ aspect of positive serial dependence could be maladaptive to low-level auditory processes.

Serial dependence, however, also occurs robustly with global processes where meaning and identity are extracted from sensory input. This is well illustrated by face perception studies showing robust serial dependence for various aspects of faces, including a face's identity (Kok et al., 2017; Liberman et al., 2014; Turbett et al., 2019,  2021), attractiveness (Taubert, Van der Burg, et al., 2016; Van der Burg et al., 2019; Xia et al., 2016), sex (Taubert, Alais, et al., 2016) and emotional expression (Liberman et al., 2018). In the current paper, we will test for serial dependence in a global aspect of auditory speech perception known as prosody, the quality of speech defined by factors such as timing, rhythm and pitch which help the listener extract meaning (Dahan, 2015). Prosody is a good candidate for serial dependence because it is fundamentally global, operating at a suprasegmental level beyond the processing of individual phoneme elements.

Not all stimulus sequences elicit positive serial dependencies. For some stimuli, there is repulsion from the previously seen stimulus, so that the difference between the current and previous stimulus is exaggerated. This effect is similar to traditional negative (also known as repulsive) aftereffects seen after sustained exposure to an adapting stimulus, such as the visual tilt (Gibson & Radner, 1937) and motion aftereffects (Anstis et al., 1998) and the auditory frequency modulation (Regan & Tansley, 1979) and timbre aftereffects (Piazza et al., 2018). In the phonetic domain, negative aftereffect are known as selective speech adaptation, first introduced by Eimas and Corbit (1973). Sequences of varying auditory frequency sweeps, for example, cause the perceived direction of a given frequency sweep to exhibit a negative dependency on the preceding one (Alais et al., 2015). Similarly, sequences of brief audio-visual stimuli varying in relative timing cause a negative shift in temporal order perception (Van der Burg et al., 2013, 2015). As with positive serial dependencies, negative dependencies may also serve a useful perceptual function, in this case by helping individuate successive stimuli and improving our sensitivity to change. Both positive and negative dependencies are thus functionally useful, and there are even examples of positive and negative dependencies arising simultaneously from different attributes of a single stimulus, as observed in motion perception (Alais et al., 2017), face perception (Taubert, Alais, et al., 2016) and phoneme identification (Vroomen et al., 2007).

## Experiment 1

**Figure 1.**
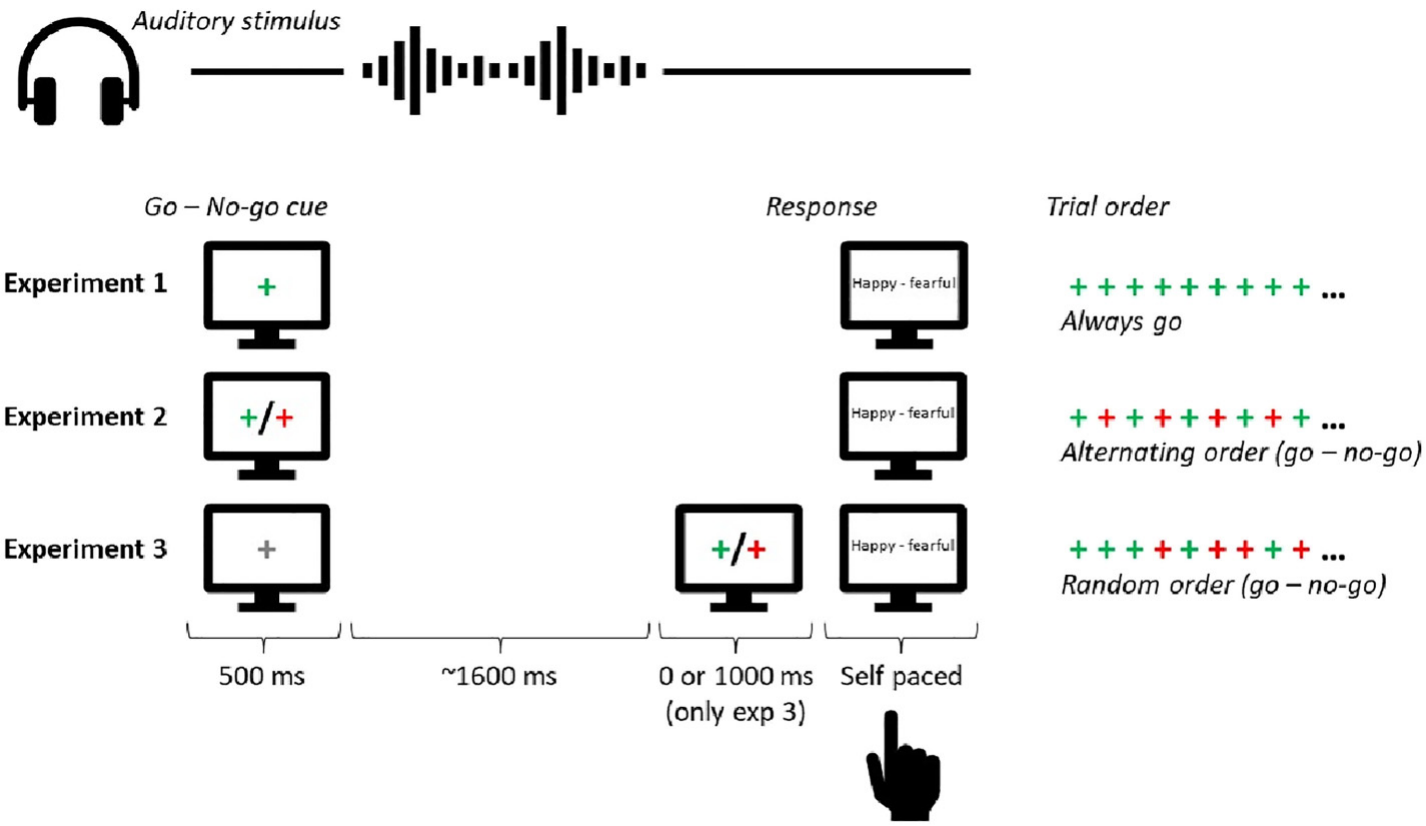
Illustration of the procedure in Experiments 1-3. Participants were either instructed to respond to the auditory stimulus (a go trial) or to withhold their response (a no-go trial). In Experiments 1 and 2, participants knew in advance whether a response on a given trial was required before the auditory stimulus appeared. In Experiment 1, participants were instructed to respond to the auditory stimulus on every trial. In Experiment 2, participants were instructed that the trial types would alternate between go-trials (respond to the auditory stimulus) and no-go trials (withhold response). A go/no-go cue was provided to remind the participant of the next trial type. In Experiment 3, participants received a neutral cue prior to the presentation of the auditory stimulus. After the auditory stimulus, participants were informed about whether a response was required or not by presenting a go or no-go cue after 0 or 1000 ms delay. The trial type (go or no-go) was randomly determined on every trial. (Colour online).

In a recent study, a negative effect was observed when participants judged the emotion of an auditory test stimulus after being exposed to auditory or audio-visual emotional stimuli during a *passive* adaptation procedure prior to the test trials (Baart & Vroomen, 2018; Bestelmeyer et al., 2010; Skuk & Schweinberger, 2013). The aim of Experiment 1 is to investigate whether the brain rapidly adapts to the emotion of a single auditory stimulus, in the absence of an explicit adaptation procedure (see, e.g., Harvey et al. (2014); Van der Burg et al. (2013) for a similar logic). Participants were exposed to spoken sentences that varied in emotional affect. More specifically, seven versions of a single sentence were presented, and prosody ranged from *happy* to *fearful* (see the *Stimuli and Apparatus* section for more details). After hearing a sentence, participants rated the emotion of the auditory stimulus on a 7-point Likert scale. Figure 1 illustrates the procedure used in Experiment 1. If the brain rapidly adapts to auditory stimuli in a negative way, such as reported by Alais et al. (2015) for frequency sweeps and by Bestelmeyer et al. (2010) for passive adaptation to auditory emotion, then we expect the perceived emotion on a given trial *t* to be contingent on the emotion of the auditory stimulus on the previous trial (*t* − 1) in a negative relationship. That is, a stimulus on a given trial *t* is perceived as more fearful when the preceding trial's emotion is happy than when it is fearful and vice versa.

### Method

#### Participants

Twenty-three Dutch-speaking participants (20 females; 3 males; the mean age was 22.1 years, ranging from 18 to 45 years) participated in the present experiment. The participants were naïve as to the purpose of the experiment and received €8 per hour or course credits for their participation. Written informed consent was obtained prior to testing. The study was conducted in accordance with the declaration of Helsinki and approved by the ethical committee from the Vrije Universiteit Amsterdam.

#### Stimuli and Apparatus

The auditory stimuli were seven versions of the Dutch sentence ‘Zijn vriendin kwam met het vliegtuig’ (‘His girlfriend arrived by plane’) that varied in emotional affect from *happy* to *fearful* (see Baart & Vroomen, 2018; Bertelson et al., 2000). The continuum was created by systematically changing (1) the fundamental frequency of the sentence, (2) the excursion size of the fundamental frequency in the accented syllables (‘vrienDIN’ and ‘VLIEGtuig’), and (3) the overall duration in the *happy* sentence (that served as source signal) in six steps towards the *fearful* endpoint using PSOLA (pitch synchronous overlap and add method; Valbret et al., 1992). The average pitch values of all continuum sentences and critical segments are provided in Table 1 (see also Figure 2 in Baart & Vroomen (2018)).

**Table 1.** Average pitch (F0) for the continuum sentences and the stressed-syllable items (syllable stress is indicated in underlined font), onset time of the final syllable and stimulus duration.

		Average F0 (Hz)			Onset last syllable (ms) ‘…Tuig’	Stimulus duration (ms)
		Full sentence	‘Vriendin’	‘Vliegtuig’		
‘Happy’	Token −3	210	248	189	1344	1755
	Token −2	221	257	198	1318	1694
	Token −1	235	269	212	1288	1660
	Token 0	249	283	221	1262	1626
	Token 1	263	296	235	1232	1541
	Token 2	276	307	245	1216	1554
‘Fearful'	Token 3	285	313	252	1190	1513

The experiment was run in a dimly lit cubicle using E-prime software. Participants sat at a distance of approximately 80 cm from the LCD monitor (120 Hz refresh rate) and wore Sennheiser headphones during the course of the experiment.

#### Design and Procedure

A trial started with the presentation of a grey fixation cross at the centre of a black screen for 500 ms. Subsequently, the auditory stimulus was presented and after the stimulus offset, participants were instructed to rate the emotional valence on a 7-point Likert scale from 1 (*happy*) to 7 (*fearful*), by pressing the corresponding key (1–7). Each of the seven continuum sentences were delivered 10 times per block in random order. In total there were 3 practice blocks of 70 trials each to familiarise participants with the task, and 8 experimental blocks of 70 trials each. Participants received instructions on the screen prior to the experiment. The next trial was initiated after participants made their response.

### Results

**Figure 2.**
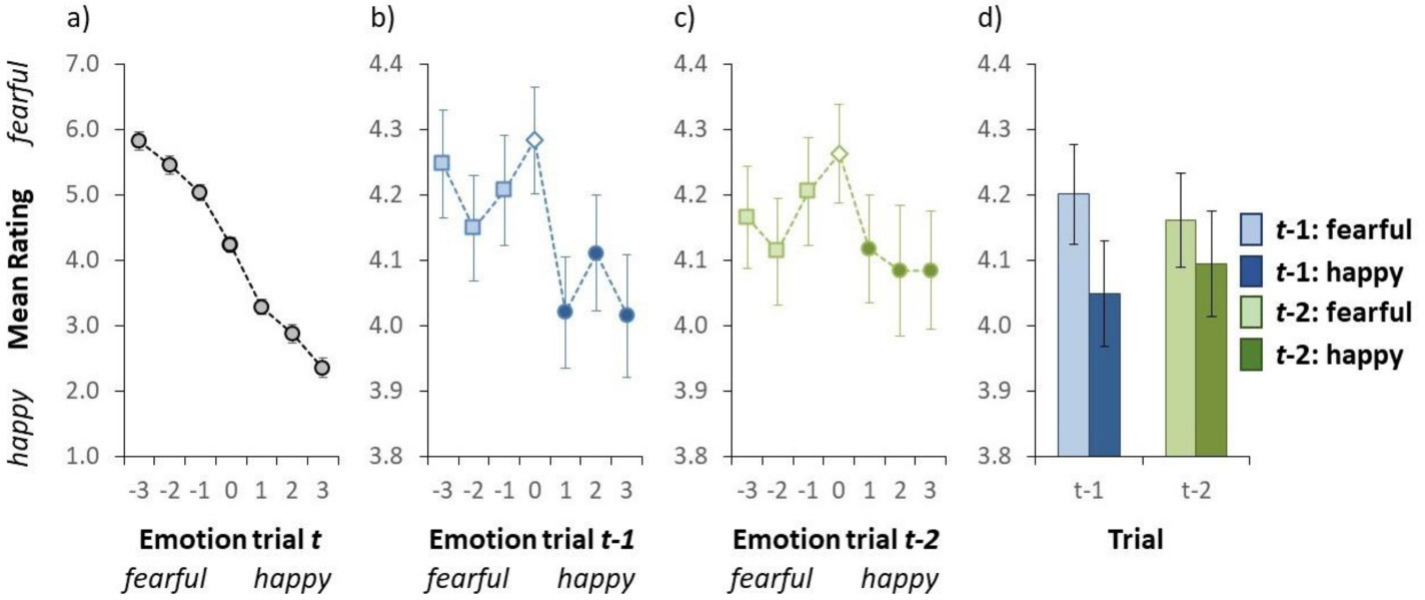
Results of Experiment 1. (a) Mean valence rating as a function of the auditory emotion on a given trial *t*. (b) Mean valence rating as a function of the auditory emotion on trial *t* − 1. (c) Mean valence rating as a function of the auditory emotion on trial *t* − 2. In panel a-c, the emotion on the *x*-axis (i.e., the actual stimulus) was either fearful (negative numbers) or happy (positive numbers). (d) Mean valence rating as a function of the auditory emotion on trial *t* − 1 (blue bars on the left) and trial *t* − 2 (green bars on the right). Here the light blue (collapsed over the light blue squares in Figure 2b) and the light green (collapsed over the light green squares in Figure 2c) bars represent a fearful emotion on trial *t* − 1 and trial *t* − 2, respectively. The dark blue (collapsed over the dark blue circles in Figure 2b) and the dark green (collapsed over the dark green circles in Figure 2c) bars signify a happy emotion on trial *t* − 1 and trial *t* − 2, respectively. In all panels, the error bars indicate ±1 standard error of the mean. (Colour online).

Practice trials and the first two trials of each block were discarded from further analyses. Furthermore, the data from three participants were excluded from further analyses as they did not perceive the emotional valence in the auditory stimuli (their rating difference between *fearful* and *happy* was < 1.5, whereas the group mean difference was 3.47; see also Baart & Vroomen, 2018, for a similar exclusion criterion). One participant swapped the response keys during the course of the experiment in a consistent fashion. For this participant we flipped the responses manually. The results from Experiment 1 are shown in Figure 2.

#### Mean Valence Ratings

Figure 2a illustrates the group-averaged valence rating for each auditory test sentence. We conducted a repeated-measures ANOVA on the mean rating with auditory sentence as within subject variable. Alpha was set to .05. Shapiro–Wilk tests were conducted to test for normality. The ANOVA yielded a statistically significant effect of the auditory test sentence, *F*(6, 114) = 128.6, *p* < .001, as the mean valence rating dropped from 5.82 to 2.35 from the *fearful*- to the *happy*-end, respectively. This result is important as it illustrates that participants were able to rate the emotion of the auditory stimulus as intended (see also Baart & Vroomen, 2018; Bertelson et al., 2000).

#### Mean Valence Ratings as a Function of the Emotion on Trial *t* − 1

An inter-trial analysis was conducted to examine whether participants rapidly adapt to the emotion of the auditory test stimulus. Figure 2b illustrates the group-averaged valence rating (collapsed over all auditory test sentences) as a function of the emotion of the auditory stimulus on the previous trial. We conducted a repeated-measures ANOVA on the mean valence rating with emotion on the preceding trial (*t* − 1) as within subject variable to examine whether the emotion on the previous trial affected the rating on the current trial. The ANOVA yielded a statistically significant effect of the emotion of the auditory stimulus on the previous trial, *F*(6, 114) = 5.034, *p* < .001, indicating that the valence rating varied as a function of the stimulus on the preceding trial. Subsequently, we investigated what was driving this serial dependence (i.e., inter-trial effect). Our goal was not to investigate the effect of each particular stimulus level on the preceding trial on a given current trial, but instead to examine whether the emotion in general (i.e., fearful or happy) on the previous trial affected performance on the current one. Based on the performance in Figure 2a, we labelled the negative stimuli as fearful stimuli, and the positive stimuli as happy. For each individual, we calculated the mean valence rating for fearful stimuli on the preceding trial (by calculating the mean valence rating over the three light blue squares in Figure 2b) and the mean valence rating for happy stimuli on trial *t* − 1 (by calculating the mean valence rating over the three dark blue circles in Figure 2b). The mean valence rating was significantly higher when the previous stimulus was fearful (4.20 Figure 2d) than when the previous stimulus was happy (4.01; see Figure 2d), *t*(19) = 3.102, *p* = .006 (two-tailed *t*-test; assumption of normality was not violated, *p* = .952). Taken together, these results indicate that the auditory stimulus on a given trial was perceived as being more fearful when the auditory stimulus was preceded by a fearful emotion than when it was preceded by a happy emotion (i.e., an assimilative effect, rather than a negative one).

**Figure 3.**
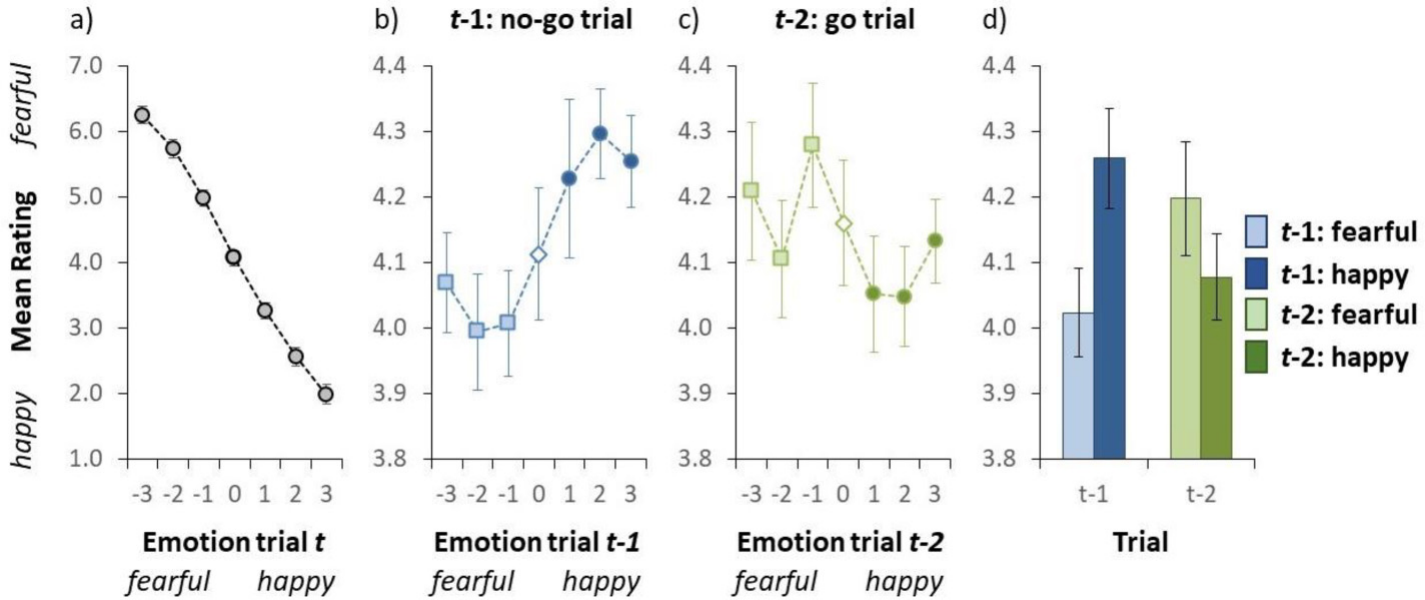
Results of Experiment 2. (a) Mean valence rating on go trials as a function of the auditory emotion on a given trial *t*. (b) Mean valence rating on go trials as a function of the auditory emotion on trial *t* − 1 (no-go trial). (c) Mean valence rating on go trials as a function of the auditory emotion on trial *t* − 2 (go trial). In panel a-c, the emotion on the *x*-axis (i.e., the actual stimulus) was either fearful (negative numbers) or happy (positive numbers). (d) Mean valence rating on go trials as a function of the auditory emotion on trial *t* − 1 (blue bars on the left) and trial *t* − 2 (green bars on the right). Here the light blue (collapsed over the light blue squares in Figure 3b) and the light green (collapsed over the light green squares in Figure 3c) bars represent a fearful emotion on trial *t* − 1 and trial *t* − 2, respectively. The dark blue (collapsed over the dark blue circles in Figure 3b) and the dark green (collapsed over the dark green circles in Figure 3c) bars signifies a happy emotion on trial *t* − 1 and trial *t* − 2, respectively. Error bars indicate ±1 standard error of the mean. (Colour online).

#### Mean Valence Ratings as a Function of the Emotion on Trial *t* − 2

A second inter-trial analysis was conducted to examine whether the valence rating on a given trial also depends on the emotion of the auditory stimulus two trials back. Figure 2c illustrates the group-averaged valence rating (collapsed over all auditory test sentences) as a function of the emotion of the auditory stimulus on trial *t* − 2. We conducted a repeated-measures ANOVA on the mean valence rating with emotion on trial *t* − 2 as within subject variable. The ANOVA yielded a statistically significant effect, *F*(6, 114) = 2.193, *p* = .049, indicating that the valence rating varied as a function of the stimulus two trials back. This effect was further examined by comparing the valence rating for fearful stimuli on trial *t* − 2 (the mean valence rating over the light green squares in Figure 2c) compared to the happy stimuli on trial *t* − 2 (the mean valence rating over the dark green circles in Figure 2c). A two-tailed *t*-test yielded a statistically significant effect, *t*(19) = 2.175, *p* = .042, indicating that the mean valence rating was significantly higher when the stimulus on two trials back was fearful (4.10 Figure 2d) than when the stimulus was happy (4.16; see Figure 2d). Note that the assumption of normality was not violated, *p* = .715. Taken together, these results indicate that the auditory stimulus on a given trial was perceived as being more fearful when trial *t* − 2 was fearful than when it was a happy emotion (again, an assimilative effect).

The results revealed that the perceived emotion on a given trial *t* depends on the emotion of the auditory stimulus on the preceding trial (*t* − 1), and also on the emotion of the auditory stimulus two trials back (*t* − 2). More specifically, and contrary to what we predicted beforehand, we observed a positive serial dependence for both cases, indicating that the perceived emotion on trial *t* was biased towards the emotion on the previous trial, and to a lesser extend to the emotion two trials back. Our finding of a positive effect, however, squares with the observation made in the Introduction that positive serial effects would be more likely to occur in audition for global stimuli such as prosody. It is unclear why we found a positive serial effect for prosody when Bestelmeyer et al. (2010) reported a negative one. Finding a positive serial dependence, however, does not necessarily imply that there was no negative serial dependence at all as some stimuli simultaneously produce positive and negative serial dependencies (Alais et al., 2017; Taubert, Alais, et al., 2016), which can sum together (Alais et al., 2017). It is possible that effects of both signs occurred but the final outcome was a positive inter-trial effect because its magnitude was larger. Another factor is that Bestelmeyer et al. (2010) used an extended *passive* adaptation procedure, whereas ours was active in that each stimulus presentation required the participant to make a judgement. This procedural difference is examined in Experiment 2 where we examine whether withholding the response on the previous trial influences the sign of the inter-trial effect.

## Experiment 2

In Experiment 2, participants were again exposed to ambiguous auditory sentences whose prosody ranged from *happy* to *fearful*. As in Experiment 1, participants were required to rate the emotion of the auditory stimulus, but not on every trial. To circumvent the possibility of a response bias (i.e., simply repeating the response on the preceding trial), we instructed participants to withhold their response on adapter trials (no-go trials) and to rate the emotion on test trials (go trials) only. Adapter and test trials were presented in alternating order so that participants were able to optimally prepare for test trials and to ignore the adapter trials (see also Van der Burg et al., 2013). Furthermore, participants were informed about the trial type as the fixation cross was green for go trials and red for no-go trials. Figure 1 illustrates the procedure applied in Experiment 2. The aim of Experiment 2 is to investigate whether the valence rating on a given test trial *t* depends on the emotion of an auditory stimulus on the preceding adapter trial even though no explicit task is required. If the brain rapidly adapts to the emotion of an auditory stimulus in an automatic fashion (see, e.g., Van der Burg et al., 2013; Van der Burg et al., 2018), then we expect that the perceived emotion on a given test trial *t* to be contingent upon the emotion of the auditory stimulus on the previous adapter trial (*t* − 1). More specifically, a contrastive effect is expected if participants rapidly adapt to the emotion of the auditory stimulus, as in the present study we minimise the possibility for a response bias as no response is required on no-go trials. That is to say, we expect that a stimulus on a trial *t* is perceived as more fearful when the emotion of the preceding trial *t* − 1 is happy than when it is fearful, or vice versa, as no response selection and/or decision was made on the previous trial. In contrast, we expect that a stimulus on a trial *t* is perceived as more fearful when the emotion of the preceding trial *t* − 2 is fearful than when it is happy, or vice versa, as participants made a decision and a response two trials back.

### Method

#### Participants

Twenty-three Dutch-speaking participants (22 females; 1 male; the mean age was 23.8 years, ranging from 18 to 58 years) participated in the present experiment. All participants were naïve to the purpose of the experiment.

Experiment 2 was similar to Experiment 1, except for the following changes. A trial started with the presentation of a red or green fixation cross for a duration of 500 ms. The fixation cross was red for adapter trials (no-go trials) and green for test trials (go trials) to inform participants about the task. On test trials, participants were instructed to rate the emotional valence on a 7-point scale from 1 (*happy*) to 7 (*fearful*), by pressing the corresponding key (as in Experiment 1). The subsequent trial was initiated after this response. The same auditory stimuli were presented on adapter trials, but the participants were instructed to withhold their response. The subsequent trial was initiated 3,000 ms after the onset of the auditory stimulus. Prior to the experiment, participants were informed that the adapter and test trials were presented in alternating order. In total there were 3 practice blocks of 70 trials each and 11 experimental blocks of 70 trials each. For each block, the first trial was always an adapter trial.

### Results

Practice trials and the first two trials of each block were discarded from further analyses. Furthermore, the data from three participants were excluded from further analyses as they did not perceive the emotional valence in the auditory stimuli (their rating difference between *fearful* and *happy* was < 1.5, whereas the group mean difference was 4.25). The ratings on go trials are shown in Figure 3.

#### Mean Valence Ratings

Figure 3a illustrates the group-averaged valence rating for each auditory test sentence. We conducted a repeated-measures ANOVA on the mean rating with auditory sentence as within subject variable. The ANOVA yielded a statistically significant effect of the auditory sentence, *F*(6, 114) = 165.6, *p* < .001, as the mean valence rating dropped from 6.25 to 1.99 from the *fearful*- to the *happy*-end, respectively. This result is consistent with Experiment 1, illustrating that participants were capable to rate the emotion of the auditory stimulus as intended.

#### Mean Valence Ratings on go Trials as a Function of the Emotion on no-go Trial *t* − 1

An inter-trial analysis was conducted to examine whether participants rapidly adapt to the emotion of the auditory test stimulus. Figure 3b illustrates the group-averaged valence rating (collapsed over all auditory test sentences) for test trials as a function of the emotion of the auditory stimulus on the previous no-go trial. We conducted a repeated-measures ANOVA on the mean valence rating with emotion on the preceding trial (*t* − 1) as within subject variable. The ANOVA yielded a statistically significant effect of the emotion of the auditory stimulus on the previous trial, *F*(6, 114) = 4.784, *p* < .001, indicating that the valence rating varied as a function of the stimulus on the preceding trial. This effect was further examined by comparing the valence rating for fearful stimuli on the preceding trial (the mean valence rating over the light blue squares in Figure 3b) compared to the happy stimuli on trial *t* − 1 (the mean valence rating over the dark blue squares in Figure 3b). A two-tailed *t*-test yielded a statistically significant effect, *t*(19) = 6.013, *p* < .001, indicating that the mean valence rating was significantly lower when the previous stimulus was fearful (4.02) than when the previous stimulus was happy (4.26; see Figure 3d). Note that the assumption of normality was violated (*p* = .032). However, a Wilcoxon rank test also revealed a significant inter-trial effect (*p* < .001). Taken together, and contrary to the results observed in Experiment 1, this indicates a negative effect in that the auditory stimulus on a given trial was perceived as being more fearful when the auditory stimulus was preceded by a happy (no-go) emotion than when it was preceded by a fearful (no-go) emotion.

#### Mean Valence Ratings as a Function of the Emotion on go Trial *t* − 2

Another analysis was conducted to examine whether the valence rating on a given test trial also depends on the emotion of the auditory stimulus 2 trials back (i.e., a go trial). Figure 3c illustrates the group-averaged valence rating (collapsed over all auditory test sentences) as a function of the emotion of the auditory stimulus on trial *t* − 2. We conducted a repeated-measures ANOVA on the mean valence rating with emotion on trial *t* − 2 as within subject variable. The ANOVA yielded a trend towards a main effect of emotion on trial *t* − 2, *F*(6, 114) = 2.124, *p* = .056. This effect was further examined by comparing the mean valence rating for fearful stimuli on trial *t* − 2 (the mean valance rating over the light green squares in Figure 3c) compared to the happy stimuli on trial *t* − 2 (the mean valance rating over the dark green circles in Figure 3c). A two-tailed *t*-test yielded a statistically significant effect, *t*(19) = 2.135, *p* = .046, as that the mean valence rating was higher when the stimulus two trials back was fearful (4.20) than when the stimulus was happy (4.08; see Figure 3d). That is, the *t* − 2 effect is positive, as found in Experiment 1. The assumption of normality was not validated (*p* = .217).

The results for Experiment 2 demonstrate that the dependency of perceived emotion on a given trial *t* can depend on preceding trials in two different ways, depending on task requirements. Figure 3b shows a negative effect (i.e., a negative serial dependency) on trials when no response was required (i.e., *t* − 1 trials). Importantly, given the experiment was designed with alternating go and no-go trials, it was clear to participants that *t* − 1 trials required no response. This is consistent with other studies showing negative effects in audition (Alais et al., 2015; Piazza et al., 2018; Regan & Tansley, 1979). In contrast, Figure 3c shows a positive effect (i.e., a positive serial dependency) on the emotion of the auditory stimulus on *t* − 2 trials, which did require a response. Why would the sign of the serial dependency reverse depending on whether the task requirement was to respond or not to respond? The vast majority of studies report either a positive or negative serial effect, but some have reported both positive and negative effects within a single experiment (see, e.g., Alais et al., 2017; Taubert, Alais, et al., 2016). In the Alais et al. study, the positive dependency was observed for the stimulus attribute that participants were instructed to respond to, and the other attribute produced a negative dependency. This suggests that both dependencies reflect different mechanisms (and they may even sum together (Alais et al., 2017). In their study and ours, it was the response that appeared to drive the positive serial dependency and other findings concur with this (Bae & Luck, 2020).

Recently, Fritsche et al. (2017) examined whether positive and negative serial dependencies reflect different processes. Participants were presented with randomly oriented Gabor patches either left or right of fixation and report the orientation. Fritsche et al. reported serial effects of perceived orientation whose sign depended on whether the *t* and *t* − 1 trials occurred at the same or different location. A negative sequential effect was observed for the same location (i.e., the classic tilt aftereffect, Gibson & Radner, 1937) whereas a positive sequential effect was observed for different locations (see also Fischer & Whitney, 2014). Fritsche account for the repulsion in terms of early, perceptual effects (i.e., adaptation) and the attraction in terms of later, post-perceptual processes (e.g., working memory, decision-making). This positive effect is assumed to integrate information over time in order to reduce noise from neural signals (Burr & Cicchini, 2014; Fischer & Whitney, 2014; Kiyonaga et al., 2017) and may also feedback to influence early perceptual processes (Cicchini et al., 2021) or may overcome negative effects due to adaptation (Sheehan & Serences, 2022).

The results of Experiment 2, then, in showing opposite serial effects depending on whether the preceding trial involved a response or not, are consistent with previous findings. The results of our go/no-go manipulation underline the importance of the response in eliciting a positive serial dependence, and the negative serial dependence that arises as kind of rapid adaptation if no response is required. Experiment 3 goes further to try to illuminate the role played by the manual response in serial dependence.

## Experiment 3

The full sequence of processes involved in completing a trial in these experiments involves three stages. There is perceptual encoding of the stimulus (which produces a negative dependence: Figure 3b), followed by the perceptual decision stage (which produces a positive dependence: Figure 2b). A final stage involves the reporting of the perceptual decision by motor response. Data suggests that the motor response also leaves a serial signature, in this case a negative serial dependence (see, e.g., Zhang & Alais, 2020). In Experiment 3, we examine whether our task produces a serial dependence due to the motor response, and whether the sign of the dependence is positive or negative. The experiment was very similar to Experiment 2, except that the order of the go and no-go trials was randomly determined instead of alternating from trial to trial. Figure 1 illustrates the procedure used in Experiment 3. Importantly, participants did not know whether a response was required until after the trial had finished (the fixation cross became red if no response was required or green if a response was required). Consequently, participants had to prepare a response for each trial in case a response was required (see Los & Van der Burg, 2010; Schuch & Koch, 2003, for a similar logic). Given that the two processes of early stimulus encoding and preparation of a response will be present in all trials, the difference between response and no-response trials will be limited to whether participants made a manual response to complete the trial. This will reveal if there is a serial dependence linked to indicating a response, and whether the sign of that dependence is positive or negative. Furthermore, in Experiment 3, we also manipulated the interval (0 ms versus 1,000 ms) between the offset of the auditory test sentence and the go/no-go signal indicating whether to respond or not to investigate claims from visual serial dependence studies that increasing the response delay increases serial dependence magnitude (Bliss et al., 2017; Fritsche et al., 2017).

### Method

#### Participants

Twenty-three Dutch-speaking participants (22 females; 1 male; the mean age was 23.8 years, ranging from 18 to 58 years) participated in the present experiment. All participants were naïve as to the purpose of the experiment.

Experiment 3 was similar to Experiment 1, except for the following changes. A trial started with the presentation of a gray fixation cross for a duration of 500 ms. Subsequently, the auditory stimulus was presented. On half of the trials, participants were asked to rate the emotion (go trial), and on the remaining trials participants were instructed to withhold their response (no-go trial). As in Experiment 2, the fixation cross became red or green to signify a no-go or go trial, respectively. However, the go or no-go signal was provided either immediately after the auditory signal (delay = 0 ms) or delayed by 1,000 ms. The next trial was initiated after participants made their response, or after 675 ms if no response was required (this interval was based on the average response time in Experiment 1). Participants performed three practice blocks of 112 trials each followed by eight experimental blocks of 112 trials each. Trial type (go vs. no-go), delay (0 vs. 1,000 ms) and the emotion of the auditory stimulus were manipulated within blocks and the order was randomly determined.

### Results

**Figure 4.**
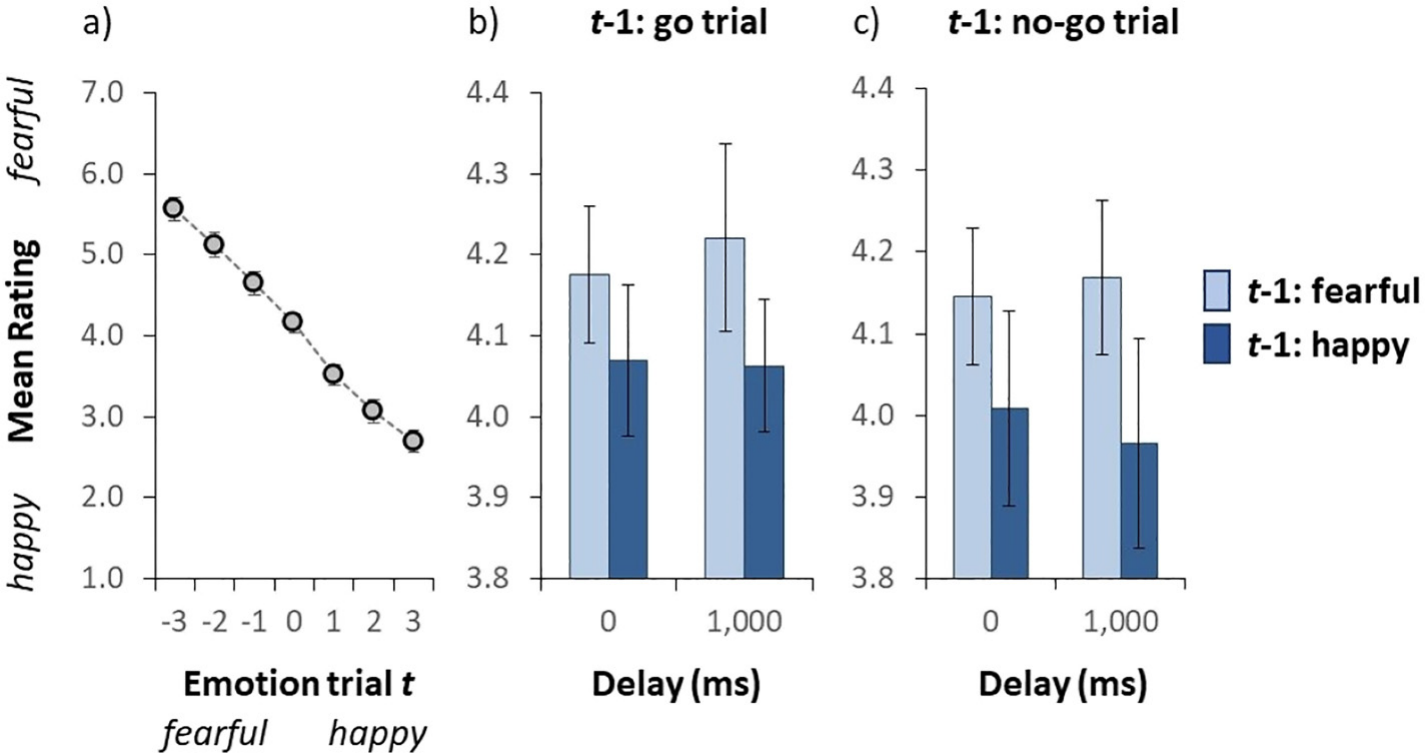
Results of Experiment 3. (a) Mean valence rating as a function of the auditory emotion on a given trial *t*. The emotion on the *x*-axis (i.e., the actual stimulus) was either fearful (negative numbers) or happy (positive numbers). (b and c) Mean valence rating (collapsed over all auditory test sentences) as a function of the emotion of the auditory stimulus on the previous trial (*t* − 1) and the delay to respond to the emotion on the preceding trial for go and no-go trials on *t* − 1, respectively. Error bars indicate the standard error of the mean. (Colour online).

Practice trials and the first two trials of each block were discarded from further analyses. Furthermore, the data from five participants were excluded from further analyses as they did not perceive the emotional valence in the auditory stimuli (their rating difference between ‘fearful’ and ‘happy’ was < 1.5, whereas the group mean difference was 2.87). The results for the remaining participants are shown in Figure 4.

#### Mean Valence Ratings

Figure 4a illustrates the group-averaged valence rating for each auditory test sentence. We conducted a repeated-measures ANOVA on the mean valence rating with auditory sentence as within subject variable. The ANOVA yielded a statistically significant effect of the auditory sentence, *F*(6,108) = 88.06, *p* < .001, as the mean valence rating dropped from 5.57 to 2.70 from the *fearful*- to the *happy*-end, respectively, again indicating that the auditory stimuli were perceived as intended.

#### Mean Valence Ratings as a Function of the Emotion on Trial *t* − 1

Figures 4b and 4c illustrate the group-averaged valence rating (collapsed over all auditory test sentences) as a function of the emotion of the auditory stimulus on the previous trial and the delay to respond to the emotion on the preceding trial for go and no-go trials on trial *t* − 1, respectively. We conducted a repeated-measures ANOVA on the mean valence rating with emotion (fearful vs. happy) and trial type (go vs. no-go) on the preceding trial and delay (0 vs. 1,000 ms) on the current trial as within subject variables. The ANOVA yielded a statistically significant effect of the emotion of the auditory test sentence on the preceding trial, *F*(1, 18) = 5.871, *p* = .026, indicating that the mean valence rating was higher when the emotion on the preceding trial was fearful (4.19) than when it was happy (4.03). Note that the assumption of normality was not violated (*p* = .363). In other words, the emotion on a given trial was biased towards the emotion on the preceding trial (i.e., a positive serial dependence). The main effect of trial type and delay failed to reach significance, *F*(1, 19) = 1.283, *p* = .272 and *F*(1, 19) = 0.032, *p* = .861, respectively. The trial type × delay interaction was not significant, *F*(1, 18) = 0.127, *p* = .726. The trial type × emotion on the previous trial interaction was not significant, *F*(1, 19) = 0.133, *p* = .720. The delay × emotion on the previous trial as well as the three-way interaction both failed to reach significance, *F*(1, 18) = 0.996, *p* = .332 and *F*(1, 19) = 0.008, *p* = .931, respectively. Taken together, the results suggest that neither trial type on the preceding trial nor the delay on the current trial influenced serial dependency.

In sum, Experiment 3 confirms a positive serial dependence for auditory emotion communicated via prosody, confirming the findings presented above in Experiment 1 (Figure 2b) and in the go trials of Experiment 2 (Figure 3c). While some authors have found evidence of a negative serial dependence for the response action (Zhang & Alais, 2020), we find no evidence of this in Experiment 3. We also found no effect of increasing the period that the response was held in working memory from 0 ms to 1,000 ms, in contrast to studies that have found holding a response in visual working memory for longer periods causes the serial effect size to increase (Bliss et al., 2017; Fritsche et al., 2017).

#### Post hoc Analysis

We conducted a post hoc analysis to investigate whether response inhibition played a significant role in the present study. It is known from the literature that reaction times (RTs) are typically greater after a no-go trial than after a go trial, if participants must prepare for the upcoming target but must withhold (or inhibit) their response when they receive a no-go cue after the presentation of the target stimulus on trial *t* − 1 (see, e.g., Los & Van der Burg, 2010; Schuch & Koch, 2003). This RT difference is known to reflect response inhibition. Even though we did not instruct the participants to respond as fast as possible, it is feasible that response inhibition plays a significant role in the present study as well. More specifically, with regard to Experiment 3, we expect participants to be slower after no-go trials than after go trials since they must prepare for the target on every trial (in case of go trial) and inhibit their response if a no-go cue appears after the stimulus presentation. A two-tailed *t*-test confirmed that the mean RT on go trials was indeed significantly greater after no-go trials (1,522 ms; standard error: ± 320 ms) than after go trials (1,189 ms; ± 266 ms), *t*(18) = 3.379, *p* = .003. Note that the normality assumption was violated (*p* < .001). However, a Wilcoxon signed-rank test also yielded a significant RT difference, *p* < .001. In contrast, when participants knew in advance whether a response was required or not, no such RT difference was observed when comparing the overall mean RT between Experiment 2 (557 ms; ± 71 ms; previous trial was always a no-go trial) with Experiment 1 (676 ms; ± 82 ms; previous trial was always a go trial), *t*(38) = 1.096, *p* = .280 (independent samples *t*-test). Note that the normality assumption was violated for the RT distribution in Experiment 2 (*p* = .011) and Experiment 1 (*p* = .009). However, a Mann–Whitney test also revealed no significant RT difference between Experiment 1 and Experiment 2, *p* = .265. The post hoc analysis revealed some more evidence that participants did not inhibit their response when they knew to withhold their response well in advance (Experiment 2). This bolsters our claim that the negative serial dependence observed in Experiment 2 reflects a perceptual process, whereas the positive serial dependence observed in all other experiments most likely reflects a perceptual and post-perceptual process that may sum together.

## General Discussion

These experiments examined serial dependence effects in auditory perception to test whether a stimulus series produces a positive or negative dependence between pairs of consecutive stimuli. Such an effect is often observed in sequences of visual stimuli and reveals that positive as well as negative effects mainly arising from prolonged exposure to a preceding stimulus (Leopold et al., 2001,  2005) there are also effects when exposures are brief (Kiyonaga et al., 2017; Pascucci et al., 2023). The evidence for serial dependence in series of brief auditory stimuli is mixed. Some studies have found negative serial effects for vocal affect (Bestelmeyer et al., 2010) and pitch sweeps (Alais et al., 2015) while others have found positive effects for judgements of duration, rate and pitch (Arzounian et al., 2017; Li et al., 2023; Motala et al., 2020). Here, investigating perceived emotion in a spoken phrase, we find a positive serial effect for perceived emotion. That is, the emotion contained on a given trial depended on the emotion in the preceding trial in a positive way: a preceding trial rated as fearful led to the current trial being rated as more fearful, and a preceding trial rated as happy led to the current trial being rated as more happy. In the following paragraphs, we discuss several important aspects of serial dependence that explain the previous mixed results.

The current results, in showing a positive serial prosody effect, contrast with an earlier result showing a negative serial effect for frequency sweeps using a very similar procedure (Alais et al., 2015). The explanation for this difference is not entirely clear, but it may well relate to the fact that the stimuli in the two studies were fundamentally different, with frequency and frequency modulation being a low-level auditory feature processed sub-cortically whereas prosody is a far more complex stimulus requiring cortical processing. Moreover, the task of assessing emotion is a far more complex one than discriminating the direction of a frequency sweep. While it is true frequency modulation is one element contributing to the perception of emotion in vocalisations (Dahan, 2015; McQueen & Dilley, 2020), prosody is based on a host of cues and is known to activate a group of cortical areas known as the Emotional Voice Areas which are principally the bilateral primary auditory cortices and superior temporal cortices (Frühholz et al., 2016; Grandjean, 2021) as well as frontal areas (Mauchand & Zhang, 2023). Prosody is also highly contextually dependent (i.e., it is suprasegmental), and so a degree of temporal integration is required, which would favour a positive serial dependence because in effect such dependencies are a kind of temporal averaging that serve to improve perception (Cicchini & Burr, 2018; Kiyonaga et al., 2017).

Our results from Experiment 1 also stand in contrast to those of Bestelmeyer et al. (2010; see also Skuk & Schweinberger, 2013). Their experiment also involved exposure to varying levels of emotion (in their case, ranging from fearful to angry) but showed a negative effect of the previous trial on the present one. A key difference is that their experiment was specifically designed to examine the effect of adaptation to a given emotional vocalisation on subsequent emotion discrimination and so they used an adaptation phase before playing the test stimulus. Adaptation consisted of four repetitions of the adaptation stimulus (i.e., a vocalisation) and with their stimulus durations averaging 0.75 s this made an average adaptation exposure of 3 s preceding each test. This means that there were two key differences between the studies. First, as our stimuli averaged ∼1.3 s, all our preceding stimuli were much shorter than theirs. Second, there was no task required of the participant in the Bestelmeyer study, as the adaptation phase was purely passive. Each of these differences is important in whether a positive serial effect will be obtained or not.

Stimulus duration is important because positive serial dependence operates over a short time-scale. Longer exposures quickly lead to negative perceptual effects rather than positive ones and, in addition, the positive effect is primarily driven by the preceding stimulus (Gekas et al., 2019). While positive serial effects can sometimes be found on the two-back trial, as observed here, and even weakly on the three-back trial in some rare cases, the effect size declines rapidly. Thus, in the Bestelmeyer study, both the adaptation period of 3 s and the fact that the stimulus was repeated four times is enough to expect a negative rather than positive effect. Another key difference is that in our Experiment 1 participants made an emotion judgement on every stimulus presentation whereas in Bestelmeyer et al. the adaptation period was passive and required no decision. This is relevant because positive serial dependence is often strongly driven by perceptual decisions and requires conscious awareness of the stimulus (Sujin Kim et al., 2020) while early sensory encoding produces negative effects (John-Saaltink et al., 2016; Pascucci et al., 2023) which can occur with visually suppressed stimuli (Blake et al., 2006). The key role of decision-making was confirmed in Experiment 2 where we included a mix of go and no-go trials (in alternating order) within the same session. In no-go trials, no response was required (this was cued before the trial) and we indeed reported a negative serial effect in this case, as in Bestelmeyer's study (see our Figure 3). The data from the go trials, on the other hand, required a response and we observed a trend towards a positive serial dependence. This underscores the importance of perceptual decisions in obtaining a positive serial effect and that removing the decision can reveal negative serial effects.

The key role played by perceptual decision-making is underscored by the results of Experiment 3. This experiment contained response and no-response trials and was essentially a randomised version of Experiment 2 (where go and no-go trials were presented in alternating order). The key difference was that participants did not know if a response was required until after the stimulus presentation (in Experiment 2 they were pre-cued) and so a decision had to be made on every trial in case a response was required. Conforming the importance of perceptual decision-making in positive serial dependence, we found positive serial effects in Experiment 3 after both kinds of trials. In addition, by using a randomly interleaved design where response trials were not known to the participant until after stimulus presentation, Experiment 3 also sheds light on the role of the motor action involved in indicating the perceptual decision. In these data, by showing the same positive serial effect for both response and no-response trials, the experiment indicates that the making of a manual response to indicate the decision had no effect on the positive serial dependence result. This is not always the case, for example, Zhang and Alais (2020) found a negative effect attributable to the motor response. However, a design such as used in Experiment 3 involving randomly interleaved, post-cued response/no-response trials is an effective way to reveal an influence of motor response.

Comparing response/no-response trials that are either pre-cued (Experiment 2) or post-cued (Experiment 3) is useful for revealing, respectively, the negative effects characteristic of early perceptual stages and the later positive effects at the decision level. This is important because one possible resolution of the puzzle that serial effects are sometimes positive and sometimes negative is that both positive and negative effects exist in parallel but that the positive effect is strong at short time-scales and then declines while the negative effect is weaker at first but grows stronger over longer time-scales. Consistent with this, some studies have shown that effects of both signs can co-occur (Alais et al., 2017; Taubert, Alais, et al., 2016) and that perception in such a case reflects the sum of the effects (Alais et al., 2017). If this were the case, eliminating the decision by using pre-cued no-go trials as in in our Experiment 2 would be expected to remove the positive serial effect and reveal an underlying (and perhaps weaker) negative serial effect. The results of Experiment 2 support this view by revealing a negative dependence (see Figure 3b). Once the decision stage is reinstated, as in the post-cued trials in Experiment 3, we see the positive serial effect return and no difference between response and no-response trials. In Experiment 3, we found no significant differences between the immediate (0 ms) and delayed (1,000 ms) response conditions. Some authors have argued that serial dependence arises from retaining a stimulus in working memory until the decision is required (Barbosa & Compte, 2020; Bliss et al., 2017; Mei et al., 2019), yet we observed no difference between the immediate and delayed response conditions.

Fritsche et al. (2017) introduced another method for disentangling positive and negative serial effects in a visual study by simply manipulating stimulus location from trial to trial. Although this separates perceptual from decisional processes, it is not suited to auditory stimuli such as those used in this study. Another disadvantage is that even in visual studies it is most useful where space plays a significant role. For instance, one cannot apply the same logic to disentangle perceptual from post-perceptual processes in serial dependences for aesthetic ratings of paintings (S. Kim et al., 2019), or for facial attractiveness (Kok et al., 2017; Liberman et al., 2014; Van der Burg et al., 2019; Xia et al., 2016) as face neurons show considerable place invariance. Fritsche et al.'s method might work in audition for stimuli separated by pitch (as fundamental to audition as space is to vision) or perhaps by using different speaker identities if the stimuli were vocalisations. Yet, not all auditory studies focus on vocal sounds. Van der Burg et al. (2021) found a positive serial dependence for auditory emotion in stimuli that were not vocalisations but were nonetheless highly affective (e.g., sounds from daily life, electric sounds, transport, music). Participants rated the valence and arousal of the sounds and found a positive serial dependence for both (e.g., higher valence ratings following a previous stimulus rated high on valence). In contrast to the current study, a positive dependence was also observed when participants were asked to withhold their response on the previous trial.

Overall, we have shown a positive serial dependence for auditory emotion communicated via prosody in brief vocalisation stimuli. This was established in Experiment 1 and replicated in the go trials of Experiment 2 and again in Experiment 3. We validate an effective methodology to disentangle perceptual from post-perceptual processes in Experiments 2 and 3 based on whether it is known pre- or post-stimulus that a response is required. This approach can be applied to the vast majority of studies investigating sequential dependencies to separate positive from negative serial dependence. Both serial effects may co-exist and serve different functions, with positive effects thought to reduce noise and stabilise perception while negative effects tend to increase discriminability of stimuli close to a prevailing input value. The negative dependence is a perceptual-level effect that occurs automatically, as some studies finding positive serial effects also report that the effect becomes a negative one after passive trials (Van der Burg et al., 2013; Van der Burg et al., 2021). In contrast, positive serial dependencies are decision-related and have been shown to vary in the same stimulus depending on the required decisional task (Van der Burg et al., 2019).

## References

[bibr1-03010066241235562] Alais D. Leung J. Van der Burg E. (2017). Linear summation of repulsive and attractive serial dependencies: Orientation and motion dependencies sum in motion perception. Journal of Neuroscience, 37, 4381–4390. 10.1523/JNEUROSCI.4601-15.2017 28330878 PMC6596560

[bibr2-03010066241235562] Alais D. Orchard-Mills E. Van der Burg E. (2015). Auditory frequency perception adapts rapidly to the immediate past. Attention, Perception & Psychophysics, 4, 896–906. 10.3758/s13414-014-0812-2 25522831

[bibr3-03010066241235562] Anstis S. Verstraten F. A. Mather G. (1998). The motion aftereffect. Trends in Cognitive Sciences, 2, 111–117. 10.1016/S1364-6613(98)01142-5 21227087

[bibr4-03010066241235562] Arzounian D. De Kerangal M. de Cheveigne A. (2017). Sequential dependencies in pitch judgments. Journal Acoustical Society of America, 142, 3047–3057. 10.1121/1.5009938 29195443

[bibr5-03010066241235562] Baart M. Vroomen J. (2018). Recalibration of vocal affect by a dynamic face. Experimental Brain Research, 236, 1911–1918. 10.1007/s00221-018-5270-y 29696314 PMC6010487

[bibr6-03010066241235562] Bae G.-Y. Luck S. J. (2020). Serial dependence in vision: Merely encoding the previous-trial target is not enough. Psychonomic Bulletin & Review, 27, 293–300. 10.3758/s13423-019-01678-7 31898266 PMC7101255

[bibr7-03010066241235562] Barbosa J. Compte A. (2020). Build-up of serial dependence in color working memory. Scientific Reports, 10, 10959. 10.1038/s41598-020-67861-2 32616792 PMC7331714

[bibr8-03010066241235562] Bertelson P. Vroomen J. De Gelder B. Driver J. (2000). The ventriloquist effect does not dependent on the direction of deliberate visual attention. Perception & Psychophysics, 62, 321–332. 10.3758/BF03205552 10723211

[bibr9-03010066241235562] Bestelmeyer P. E. G. Rouger J. DeBruine L. M. Belin P. (2010). Auditory adaptation in vocal affect perception. Cognition, 117, 217–223. 10.1016/j.cognition.2010.08.008 20804977

[bibr10-03010066241235562] Blake R. Tadin D. Sobel K. V. Raissian T. A. Chong S. C. (2006). Strength of early visual adaptation depends on visual awareness. Proceedings of the National Academy of Sciences, 103, 4783–4788. 10.1073/pnas.0509634103 PMC1400587 16537384

[bibr11-03010066241235562] Bliss D. P. Sun J. J. D'Esposito M. (2017). Serial dependence is absent at the time of perception but increases in visual working memory. Scientific Reports, 7, 14739. 10.1038/s41598-017-15199-7 29116132 PMC5677003

[bibr12-03010066241235562] Burr D. Cicchini G. M. (2014). Vision: Efficient adaptive coding. Current Biology, 24, R1096–R1098. 10.1016/j.cub.2014.10.002 PMC5040502 25458222

[bibr13-03010066241235562] Cicchini G. M. Benedetto A. Burr D. C. (2021). Perceptual history propagates down to early levels of sensory analysis. Current Biology, 31, 1245–1250.e2. 10.1016/j.cub.2020.12.004 33373639 PMC7987721

[bibr14-03010066241235562] Cicchini G. M. Burr D. C. (2018). Serial effects are optimal. Behavioural Brain Sciences, 41, e229. 10.1017/S0140525X18001395 30767801

[bibr15-03010066241235562] Dahan D. (2015). Prosody and language comprehension. Wiley Interdisciplinary Reviews: Cognitive Science, 6, 441–452. 10.1002/wcs.1355 26267554

[bibr16-03010066241235562] Eimas P. D. Corbit J. D. (1973). Selective adaptation of linguistic feature detectors. Cognitive Psychology, 4, 99–109. 10.1016/0010-0285(73)90006-6

[bibr17-03010066241235562] Fischer J. Whitney D. (2014). Serial dependence in visual perception. Nature Neuroscience, 17, 738–743. 10.1038/nn.3689 24686785 PMC4012025

[bibr18-03010066241235562] Fritsche M. Mostert P. de Lange F. P. (2017). Opposite effects of recent history on perception and decision. Current Biology, 27, 590–595. 10.1016/j.cub.2017.01.006 28162897

[bibr19-03010066241235562] Frühholz S. Trost W. Kotz S. A. (2016). The sound of emotions—towards a unifying neural network perspective of affective sound processing. Neuroscience & Biobehavioral Reviews, 68, 96–110. 10.1016/j.neubiorev.2016.05.002 27189782

[bibr20-03010066241235562] Fründ I. Wichmann A. Macke J. H. (2014). Quantifying the effect of intertrial dependence on perceptual decisions. Journal of Vision, 14, 9. 10.1167/14.7.9 24944238

[bibr21-03010066241235562] Gekas N. McDermott K. C. Mamassian P. (2019). Disambiguating serial effects of multiple timescales. Journal of Vision, 19, 24–24. 10.1167/19.6.24 31251808

[bibr22-03010066241235562] Gibson J. J. Radner M. (1937). Adaptation, after-effect and contrast in the perception of tilted lines. I. Quantitative Studies. Journal of Experimental Psychology, 20, 453–467. 10.1037/h0057585

[bibr23-03010066241235562] Grandjean D. (2021). Brain networks of emotional prosody processing. Emotion Review, 13, 34–43. 10.1177/1754073919898522

[bibr24-03010066241235562] Harvey C. Van der Burg E. Alais D. (2014). Rapid temporal recalibration occurs crossmodally without stimulus specificity but is absent unimodally. Brain Research, 1585, 120–130. 10.1016/j.brainres.2014.08.028 25148705

[bibr25-03010066241235562] John-Saaltink E. S. Kok P. Lau H. C. De Lange F. P. (2016). Serial dependence in perceptual decisions is reflected in activity patterns in primary visual cortex. Journal of Neuroscience, 36, 6186–6192. 10.1523/JNEUROSCI.4390-15.2016 27277797 PMC6604889

[bibr26-03010066241235562] Kim S. Burr D. Alais D. (2019). Attraction to the recent past in aesthetic judgments: A positive serial dependence for rating artwork. Journal of Vision, 19, 19. 10.1167/19.12.19 31627213

[bibr27-03010066241235562] Kim S. Burr D. Cicchini G. M. Alais D. (2020). Serial dependence in perception requires conscious awareness. Current Biology, 30, R257–R258. 10.1016/j.cub.2020.02.008 32208145

[bibr28-03010066241235562] Kiyonaga A. Scimeca J. M. Whitney D. (2017). Serial dependence across perception, attention, and memory. Trends in Cognitive Sciences, 21, 493–497. 10.1016/j.tics.2017.04.011 28549826 PMC5516910

[bibr29-03010066241235562] Kok R. Taubert J. Van der Burg E. Rhodes G. Alais D. (2017). Face familiarity promotes stable identity recognition: Exploring face perception using serial dependence. Royal Society Open Science, 4, 160685. 10.1098/rsos.160685 28405355 PMC5383812

[bibr30-03010066241235562] Leopold D. A. O'Toole A. J. Vetter T. Blanz V. (2001). Prototype-referenced shape encoding revealed by high-level after-effects. Nature Neuroscience, 4, 89–94. 10.1038/82947 11135650

[bibr31-03010066241235562] Leopold D. A. Rhodes G. Muller K. M. Jefferey L. (2005). The dynamics of visual adaptation to faces. Proceedings of the Royal Society B: Biological Sciences, 272, 897–904. 10.1098/rspb.2004.3022 PMC1564098 16024343

[bibr32-03010066241235562] Li B. Wang B. Zaidel A. (2023). Modality-specific sensory and decisional carryover effects in duration perception. BMC biology, 21, 1–17. 10.1186/s12915-022-01498-7 36882836 PMC9993637

[bibr33-03010066241235562] Liberman A. Fischer J. Whitney D. (2014). Serial dependence in the perception of faces. Current Biology, 24, 2569–2574. 10.1016/j.cub.2014.09.025 25283781 PMC4254333

[bibr34-03010066241235562] Liberman A. Manassi M. Whitney D. (2018). Serial dependence promotes the stability of perceived emotional expression depending on face similarity. Attention, Perception, & Psychophysics, 80, 1461–1473. 10.3758/s13414-018-1533-8 29736808

[bibr35-03010066241235562] Los S. A. Van der Burg E. (2010). The origin of switch costs: Task preparation or task application? Quarterly Journal of Experimental Psychology, 63, 1895–1915. 10.1080/17470211003651849 20401813

[bibr36-03010066241235562] Manassi M. Liberman A. Chaney W. Whitney D. (2017). The perceived stability of scenes: Serial dependence in ensemble representations. Scientific Reports, 7, 1971. 10.1038/s41598-017-02201-5 PMC5434007 28512359

[bibr37-03010066241235562] Mauchand M. Zhang S. (2023). Disentangling emotional signals in the brain: An ALE meta-analysis of vocal affect perception. Cognitive, Affective, & Behavioral Neuroscience, 23, 17–29. 10.3758/s13415-022-01030-y 35945478

[bibr38-03010066241235562] McQueen J. M. Dilley L. (2020). Prosody and spoken-word recognition. In C. Gussenhoven & A. Chen (Eds.), *The Oxford handbook of language prosody* (pp. 509–521). Oxford University Press. 10.1093/oxfordhb/9780198832232.001.0001

[bibr39-03010066241235562] Mei G. Chen S. Dong B. (2019). Working memory maintenance modulates serial dependence effects of perceived emotional expression. Frontiers in Psychology, 10, 1610. 10.3389/fpsyg.2019.01610 31354595 PMC6637952

[bibr40-03010066241235562] Motala A. Zhang H. Alais D. (2020). Auditory rate perception displays a positive serial dependence. i-Perception, 11, 2041669520982311. 10.1177/2041669520982311 33425315 PMC7758668

[bibr41-03010066241235562] Pascucci D. Tanrikulu ÖD Ozkirli A. Houborg C. Ceylan G. Zerr P. Rafiei M. Kristjánsson Á (2023). Serial dependence in visual perception: A review. Journal of Vision, 23, 9. 10.1167/jov.23.1.9 PMC9871508 36648418

[bibr42-03010066241235562] Piazza E. A. Theunissen F. E. Wessel D. Whitney D. (2018). Rapid adaptation to the timbre of natural sounds. Scientific Reports, 8, 13826. 10.1038/s41598-018-32018-9 30218053 PMC6138731

[bibr43-03010066241235562] Regan D. Tansley B. (1979). Selective adaptation to frequency-modulated tones: Evidence for an information-processing channel selectively sensitive to frequency changes. The Journal of the Acoustical Society of America, 65, 1249–1257. 10.1121/1.382792 458046

[bibr44-03010066241235562] Schuch S. Koch I. (2003). The role of response selection for inhibition of task sets in task shifting. Journal of Experimental Psychology: HUman Perception and Performance, 29, 92–105. 10.1037/0096-1523.29.1.92 12669750

[bibr45-03010066241235562] Sheehan T. C. Serences J. T. (2022). Attractive serial dependence overcomes repulsive neuronal adaptation. PLoS Biology, 20, e3001711. 10.1371/journal.pbio.3001711 PMC9447932 36067148

[bibr46-03010066241235562] Skuk V. G. Schweinberger S. R. (2013). Adaptation aftereffects in vocal emotion perception elicited by expressive faces and voices. PLoS One, 8, e81691. 10.1371/journal.pone.0081691 PMC3827484 24236215

[bibr47-03010066241235562] Taubert J. Alais D. Burr D. (2016). Different coding strategies for the perception of stable and changeable facial attributes. Scientific Reports, 6, 32239. 10.1038/srep32239 27582115 PMC5007489

[bibr48-03010066241235562] Taubert J. Van der Burg E. Alais D. (2016). Love at second sight: Sequential dependence of facial attractiveness in an on-line dating paradigm. Scientific Reports, 6, 1–5. 10.1038/s41598-016-0001-8 26986828 PMC4795074

[bibr49-03010066241235562] Turbett K. Palermo R. Bell J. Burton J. Jeffery L. (2019). Individual differences in serial dependence of facial identity are associated with face recognition abilities. Scientific Reports, 9, 1–12. 10.1038/s41598-019-53282-3 31792249 PMC6888837

[bibr50-03010066241235562] Turbett K. Palermo R. Bell J. Hanran-Smith D. A. Jeffery L. (2021). Serial dependence of facial identity reflects high-level face coding. Vision Research, 182, 9–19. 10.1016/j.visres.2021.01.004 33578076

[bibr51-03010066241235562] Valbret H. Moulines E. Tubach J. P. (1992). Voice transformation using PSOLA technique. Speech Communication, 11, 175–187. 10.1016/0167-6393(92)90012-V

[bibr52-03010066241235562] Van der Burg E. Alais D. Cass J. (2013). Rapid recalibration to audiovisual asynchrony. Journal of Neuroscience, 33, 14633–14637. 10.1523/JNEUROSCI.1182-13.2013 24027264 PMC6705173

[bibr53-03010066241235562] Van der Burg E. Alais D. Cass J. (2015). Audiovisual temporal recalibration occurs independently at two different time scales. Scientific Reports, 5, 14526. 10.1038/srep14526 26455577 PMC4600976

[bibr54-03010066241235562] Van der Burg E. Alais D. Cass J. (2018). Rapid recalibration to audiovisual asynchrony follows the physical—not the perceived—temporal order. Attention, Perception & Psychophysics, 80, 2060–2068. 10.3758/s13414-018-1540-9 29968078

[bibr55-03010066241235562] Van der Burg E. Rhodes G. Alais D. (2019). Positive sequential dependency for face attractiveness perception. Journal of Vision, 19, 6. 10.1167/19.12.6 31621804

[bibr56-03010066241235562] Van der Burg E. Toet A. Brouwer A.-M. Van Erp J. B. F. (2021). Serial dependence of emotion within and between stimulus sensory modalities. Multisensory Research, 1, 1–22. 10.1163/22134808-bja10064 34592713

[bibr57-03010066241235562] Vroomen J. van Linden S. De Gelder B. Bertelson P. (2007). Visual recalibration and selective adaptation in auditory–visual speech perception: Contrasting build-up courses. Neuropsychologia, 45, 572–577. 10.1016/j.neuropsychologia.2006.01.031 16530233

[bibr58-03010066241235562] Xia Y. Yamanashi Leib A. Whitney D. (2016). Serial dependence in the perception of attractiveness. Journal of Vision, 16, 28. 10.1167/16.15.28 PMC5214899 28006077

[bibr59-03010066241235562] Zhang H. Alais D. (2020). Individual difference in serial dependence results from opposite influences of perceptual choices and motor responses. Journal of Vision, 20, 2–2. 10.1167/jov.20.8.2 PMC7438638 32744618

